# Oxidative Stress Damage as a Detrimental Factor in Preterm Birth Pathology

**DOI:** 10.3389/fimmu.2014.00567

**Published:** 2014-11-12

**Authors:** Ramkumar Menon

**Affiliations:** ^1^Department of Obstetrics and Gynecology, School of Medicine, The University of Texas Medical Branch, Galveston, TX, USA

**Keywords:** oxidative stress, preterm birth, premature rupture of fetal membranes, inflammation, oxidative damage, senescence, senescence-associated secretory phenotype

## Abstract

Normal term and spontaneous preterm births (PTB) are documented to be associated with oxidative stress (OS), and imbalances in the redox system (balance between pro- and antioxidant) have been reported in the maternal–fetal intrauterine compartments. The exact mechanism of labor initiation either at term or preterm by OS is still unclear, and this lack of understanding can partially be blamed for failure of antioxidant supplementation trials in PTB prevention. Based on recent findings from our laboratory, we postulate heterogeneity in host OS response. The physiologic (at term) and pathophysiologic (preterm) pathways of labor are not mediated by OS alone but by OS-induced damage to intrauterine tissues, especially fetal membranes of the placenta. OS damage affects all major cellular elements in the fetal cells, and this damage promotes fetal cell senescence (aging). The aging of the fetal cells is predominated by p38 mitogen activated kinase (p38MAPK) pathways. Senescing cells generate biomolecular signals that are uterotonic, triggering labor process. The aging of fetal cells is normal at term. However, aging is premature in PTB, especially in those PTBs complicated by preterm premature rupture of the membranes, where elements of redox imbalances and OS damage are more dominant. We postulate that fetal cell senescence signals generated by OS damage are likely triggers for labor. This review highlights the mechanisms involved in senescence development at term and preterm by OS damage and provides insight into novel fetal signals of labor initiation pathways.

The World Health Organization recently estimated the global preterm birth (PTB) rate for singleton gestation at 9.6% (1). The PTB rate has increased in the United States by as much as 30% during the last 25 years despite advances in medical care (1, 2). Twenty-eight percent of all neonatal deaths (deaths within the first 7 days of life) that are not related to congenital malformations are due to PTB (1, 2). The most common phenotype of PTB is *spontaneous* PTB of unknown etiology. Approximately 60% of PTBs are spontaneous, and 30–40% of these are preceded by preterm premature rupture of the fetal membranes (pPROM) (3–10). The current management of preterm labor and pPROM is based largely on inhibiting uterine contractions (7, 11–25). This approach has not been successful, as such interventions are usually performed too late in the process to succeed. A second problem with the current management of preterm labor is that only women who have clear risk factors (abnormally short cervixes) or a history of PTBs are targeted for interventions designed to prevent PTB (26–30). The vast majority of PTBs occur in women who are considered low-risk because they are either on their first pregnancies or have only had term births previously (31–37). Although the rate of PTB is lower in these women (3–5%), they make up the largest volume of clinical practice. Simple interventions that can be applied to this group are likely to have the largest impact on PTB rates. Knowledge gaps in current literature about causality and causally linked pathways make it difficult to provide appropriate or personalize interventions based on the specific risk profile of an individual (6).

Risk factors of PTB and pPROM can be classified into two major categories, static and dynamic. As shown in Figure 1, all the risk factors outlined in the outermost layer can be called static risk factors as they are unlikely to change during the course of pregnancy. Independently or in combination, these static risk factors can either predispose or cause the dynamic risk factors that are commonly diagnosed as clinical risks or pathologies associated with adverse pregnancy outcomes. Epigenetic changes that are independent of DNA base variations generated by complex interactions between various risk factors during pregnancy can also contribute to dynamic clinical risks by altering expression of certain genes. These changes can transition between static and dynamic risks. Static and dynamic risk factors produce pathways and pathophysiologies depicted in the inner circle with a unique biomarker profile contributing to labor-inducing changes, resulting in PTB or pPROM. The final effector pathways culminating in labor and delivery include inflammation and oxidative stress (OS). In normal pregnancies, these are generated by various fetal and maternal factors that signal the end of pregnancy. In PTB, the maternal–fetal signals and their causal origins are still unclear as they arise from complex etiologies and redundant pathways.

**Figure 1:**
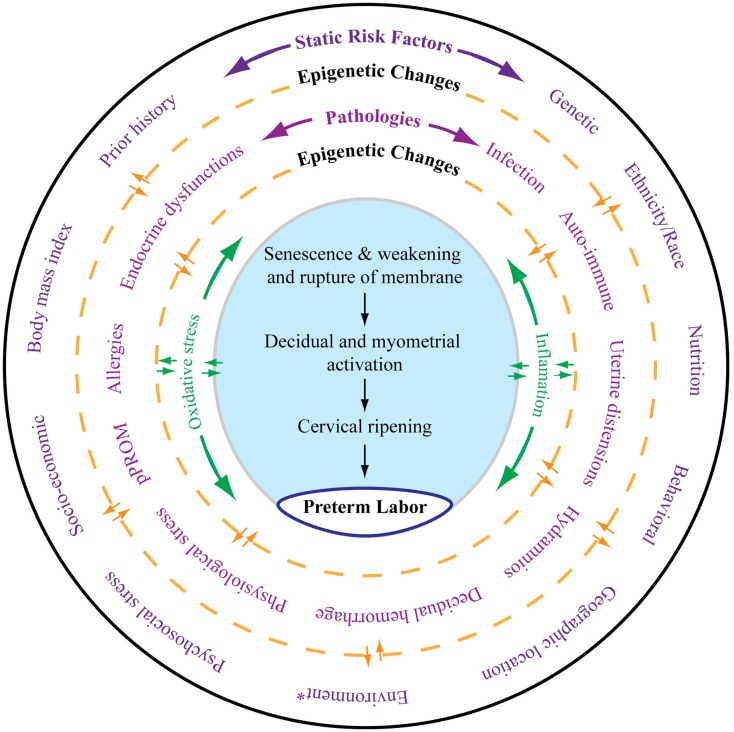
**As depicted in this figure, preterm labor (the innermost circle) is an end result of multitudes of complex interacting pathologies and pathophysiologic pathways**. The external layer (the outermost circle) shows static risk factors, including epidemiologic and genetic risk factors, that can lead to multiple disease processes as depicted in the middle circle. Epigenetic markers can be dynamic, and that complex interaction between the host environment and risk factors can produce epigenetic changes, which can lead to diseases contributing to final effecter pathways (the blue shaded area). Various diseases may also cause epigenetic changes in the genes of preterm labor pathways. Spontaneous preterm labor leading to preterm birth is a complex syndrome comprising multiple diseases, each of which can be independent initiators of labor-inducing pathways. All these disease processes can trigger inflammation and oxidative stress (OS). However, the extent and biomolecular characteristics of inflammation and OS are dependent on the type of risk that initiated the process, their complex interactions with the genetic–epigenetic system, and the disease that they cause. The final terminal pathways that involve inflammation and OS trigger labor-inducing signals from fetal membranes and decidua and cause cervical ripening, myometrial contractions, and membrane rupture, resulting in preterm labor and delivery. *Any exogenous factor that can impact pregnancy outcomes. Many of the static risk factors in the outer circles are also called environments.

Inflammation is a well-studied pathophysiology of both PTB and pPROM (38–40). This review is an attempt to shed some light on one of the under-studied mechanistic pathways: OS damage to intrauterine tissues and how it may impact pregnancy outcomes.

## Inflammation, a Well-Documented Feature of Labor and Delivery

Labor and delivery at term and preterm have an underlying pathophysiology marked by inflammatory mediators. Preterm labor is hypothesized to be driven by overwhelming inflammation that eclipses fetal uterotonic signals of organ maturity. Approximately 50% of PTBs and 70% of pPROMs are associated with intra-amniotic infection (IAI) and inflammation. Histological and microbiological findings indicate that focal infection and inflammation may play a significant role in the pathogenesis of PTB and pPROM. Inflammatory changes that precede PTB – such as leukocyte activation, increased inflammatory cytokines and chemokines, and collagenolysis of the extracellular matrix metalloproteinases (MMPs), resulting in loss of membrane structural integrity, myometrial activation, and cervical ripening – are well documented by various experimental and clinical studies (9, 38, 41). Recent studies have reported that the heterogeneity in the inflammatory response (cytokines/chemokines, toll-like-receptors, and their interactions) is associated with IAI and PTB risk factors (42–45). PTB and pPROM are well-documented host response diseases in which overwhelming immune activation can trigger labor-associated changes. The biomolecular markers that trigger labor-associated changes are different, and the difference is attributed to both epidemiologic and clinical risk factors.

## Significance of OS and Oxidative Damage

Of these risk factors, IAI, behavioral factors (cigarette smoking, alcohol intake, and drug use), obesity, malnutrition or antioxidant-deficient diets, physiologic and psycho-social stressors, environmental pollutants, genotoxic agents, and geographic location are some of the most common and well-studied risk factors of both PTB and pPROM (3, 10). The role of inflammation induced by these risk factors in PTB pathophysiology is well studied and multiple biomarkers are reported to be associated with the adverse outcome. OS, characterized by generation of reactive oxygen species (ROS), is an inseparable component of inflammation. Generation of ROS as a part of the aerobic energy building process is inevitable and is well balanced (redox status) by an array of enzymatic and non-enzymatic antioxidant systems (46–50). Redox balance is maintained through the production and subsequent elimination of ROS. Cells are able to protect themselves against OS by the finely tuned regulation of redox status through endogenous enzymes, antioxidants, and other cellular mechanisms. At low levels, ROS, often generated in biological systems, is essential for cell division and survival, cell signaling, inflammation and immune functions, autophagy, and stress response (50–52). However, an overwhelming redox imbalance compromises a biological system’s ability to detoxify these highly reactive molecules or to repair any damage caused by them (53). Therefore, the former is termed “OS” and the latter “oxidative damage” (see Glossary) (54). Oxidative damage due to ROS generation has been linked to the development of adult diseases, including cardiovascular disease, cancer, chronic inflammation, and neurologic disorders. The two main sources of ROS in human cells are: (1) mitochondrial and (2) non-mitochondrial. Mitochondria generate ROS as a byproduct of the electron transport chain during respiration, and the rate of ROS production is proportional to the rate of mitochondrial respiration. The ROS production rate is thus higher when metabolic rates are high (55, 56). A vast majority of ROS in the human body is produced by mitochondria, and mitochondria are the primary sources of superoxide production other than phagocytic cells during innate immune defense.

**Figure 2:**
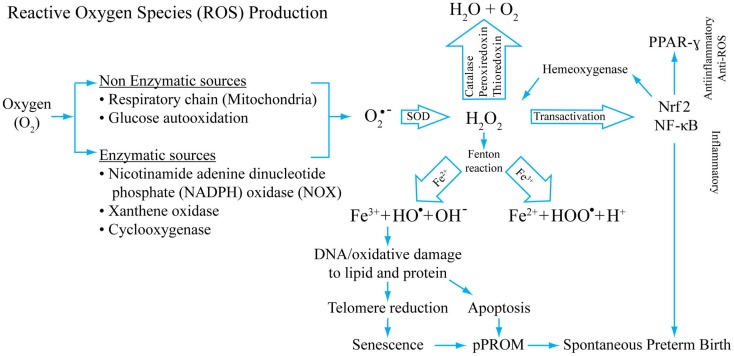
**A model of reactive oxygen production, its inactivation by antioxidant systems, and potential consequences of redox imbalances**. Superoxide radicals are generated during normal cellular respiration (mitochondrial) and also during infection following phagocytosis. Non-enzymatic and enzymatic mechanisms generate super oxide radicals (O_2_− from O_2_) that are converted into H_2_O_2_ by superoxide-dismutase (SOD). H_2_O_2_, a reactive oxygen species (ROS), is reduced to water by several different enzymes, mainly by catalase. Hydroxyl and/or superoxide radicals are created from H_2_O_2_ by a Fenton reaction that includes Fe^3+^. H_2_O_2_ is a potential transactivator and can activate a master transcription factor NF-κB, resulting in activation of several proinflammatory genes including many uterotonic genes. The Hydroxyl and/or superoxide radicals can cause DNA, protein, and lipid damage. DNA damage as detailed in this review can lead to telomere reduction and senescence. DNA damage of the cell can also cause apoptosis. One of the transactivator proteins is a nuclear factor of erythroid 2-related factor 2 (Nrf2). When activated in response to ROS, this protein can transactivate Hemeoxygenase-1 that can also neutralize H_2_O_2_, providing a regulatory mechanism. Our studies have shown that Nrf2 can cause anti-inflammatory PPAR-γ activation. Depending on the dose and type of ROS, inflammatory and/or anti-inflammatory/antioxidant pathways may be initiated. This model shows that OS-associated pathologic events result in spontaneous preterm birth and pPROM. This is not a universal model for all OS-related pathologies. Damage to cellular elements has been well documented in cancers and other diseases.

Figure 2 depicts the mechanism of ROS generation, antioxidant system, and potential damage by ROS that can cause damage during pregnancy complications. The figure also models the potential of ROS in generating inflammation and damage to cellular elements that can generate pathways leading to pPROM or PTB. This figure represents only a few key mediators of ROS generation and does not include all known endogenous antioxidants.

## Heterogeneity in Antioxidant Functions

Generation of ROS is tightly regulated by an array of enzymatic and non-enzymatic ways to keep redox balance. This balance in humans is maintained by a well-balanced antioxidant system that is responsible for homeostatic balance, which maintains physiologic functions but prevents oxidative damage. Antioxidants, in general, are substances that decrease the severity of OS by forming less active radicals or by quenching damage created by free-radical chain reactions. They can slow down or prevent damage to body cells and lower the risk of infection by improving immune function. Antioxidants can be classified into three major categories: (1) low molecular antioxidants [glutathione (GSH), vitamins C and E, bilirubin, and urate], (2) enzymes that neutralize free radicals [superoxide-dismutase, catalase, glutathione peroxidase (GPx), DT diaphorase, and peroxiredoxin], and (3) non-enzymatic proteins (thioredoxin, glutaredoxin, and metllothionines). Antioxidants can also be classified as primary and secondary. Primary antioxidants are endogenous enzymes [e.g., super oxide dismutase (SOD), catalase, and GPx] acting at the site where free radicals are formed. Secondary antioxidants are exogenous molecules obtained through food (e.g., Vitamins A, C, E, carotenoids). As detailed in Table 1, regardless of primary or secondary, endogenous or exogenous status, antioxidants have specific functions.

**Table 1:** **Antioxidants and their potential roles associated with reactive oxygen species (ROS) mediated damage**.

Antioxidants	Potential role
Super oxide dismutase (SOD)	Direct protection against ROS
Glutathione peroxidase (GPx)	
Catalase (Cat)	
Peroxiredoxin	
Glutathione	Non-specific reduction
Vitamin C	
Vitamin E	Protection against lipid peroxidation
β-Carotene	
Transferrin	Metal sequestration
Lactoferrin	
Ferritin	
Metallothionein (MT)	
DNA repair enzymes	Repair enzymes
Macroxyproteinases	
Glutathione transferase (GST)	
Thioredoxins (Trx)	

## Why Antioxidant Supplementation Fails in Preventing Adverse Pregnancy Outcomes?

A healthy pregnancy is characterized by a stable balance between ROS and antioxidants (46, 47, 49). Redox imbalance is an underlying pathologic feature of many pregnancy complications. All of the PTB and pROM risk factors detailed above are capable of causing redox imbalance, leading to the production of superoxide, hydrogen peroxide, hydroxyl ions, and nitric oxide that can damage collagen matrix and consume antioxidant defenses. These events can trigger uterine contractions (labor), leading to PTB. Based on this imbalance theory, antioxidant clinical trials have been primarily carried out to compensate for deficiencies and to maintain redox balances. However, these trials expected to reduce OS and improve pregnancy outcomes have met with minimal success (36, 57–61). Numerous reasons can be cited for the failure of these trials, but the following primary factors are highlighted:

1. OS-induced tissue injury or damage is the likely pathology and trigger of several downstream effects like initiation of labor or membrane rupture. OS damage generates a vicious cycle of events (enhanced inflammation, cell death, and tissue destruction), and antioxidants do not regulate these events and are unlikely to impact OS damage. Therefore, it is unlikely that reversing OS through antioxidant supplementation would reduce the risk of adverse pregnancy outcomes.
2. Clinical trials are conducted based on the generalized theory that OS is an underlying pathology. This theory is unlikely; this may be the case for a limited subset of subjects with specific exposures with ROS as an initiator of downstream events.
3. Biomarker screenings for ROS are not done prior to intervention to determine the type of ROS or to assess the amount of ROS-associated damage. Even the type of biomarker measures may not be reflective of risk exposure or the extent of ROS because these biomarkers may represent an end stage indicative of tissue damage and not necessarily risk.
4. Like inflammation, ROS is not a homogeneous phenomenon. Different tissues and cells, cytoplasmic and nuclear membranes, and the contents of different organelles (inner and outer membranes of mitochondria) may react differently to various stressors, producing distinct patterns of ROS. The antioxidants used in trials may curtail a particular ROS pathway or production of a specific free radical, but depending on the type of risk factor (e.g., cigarette smoke vs. poor nutrition), the ROS pathway and antioxidant requirement may be different.
5. Reactive oxygen species-associated damage can be any of the major functional elements mentioned earlier, and the degree of damage is likely dependent on the site, risk factors, and types of triggers that initiate an ROS-mediated response.

## Ambiguity in Antioxidant and OS Damage Marker Analysis

**Figure 3:**
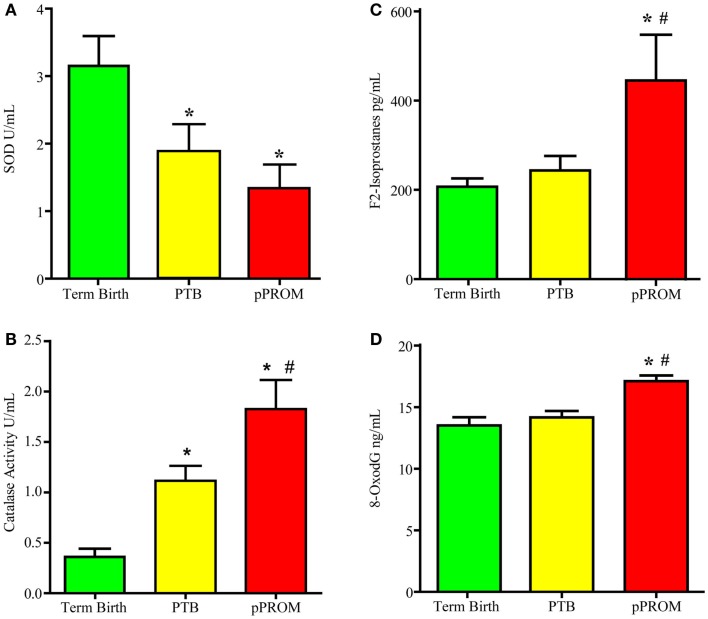
**Maternal plasma levels of two antioxidants super oxide dismutase (SOD) and catalase and two OS markers, 8-oxodG and F2-Isoprostanes**. **(A)** Antioxidant enzyme Super oxide dismutase concentrations. **(B)** Antioxidant enzyme catalase concentrations. **(C)** Lipid peroxidation product F2-Isoprostane concentration. **(D)** bDNA oxidation product 8-oxo-7,8-dihydro-2’-deoxyguanosine (8-oxodG) concentration. *P < 0.05 compared to term birth; ^#^*P* < 0.05 compared to PTB.

Many studies have reported antioxidant deficiencies as a causal factor associated with adverse pregnancy outcomes. In the wake of a failed antioxidant supplementation trial, my laboratory conducted a cross-sectional study examining two antioxidant enzymes (SOD1 and catalase) and two OS damage markers (F2-Isoprostane – lipid peroxidation product – and 8-OxodG – DNA damage). Banked maternal plasma samples were collected from subjects at the time of delivery admission for either a term labor (*n* = 19), preterm labor (*n* = 18), or pPROM (*n* = 13). An ELISA-based analysis was performed using these samples. If each of the markers is analyzed independently, multitudes of interpretations can be derived. For example, if SOD was the only analyte tested, as shown in Figure 3A, SOD was higher at term and reduced significantly in PTB and pPROM. These data are normally interpreted as heightened OS at pPROM, followed by PTB, and none at term birth. If catalase was the only marker analyzed using the same samples (Figure 3B), term birth has the highest OS, followed by PTB, and none at pPROM, which highlights the ambiguity associated with singe marker analysis of OS. Even examining the two enzymes together only provides conflicting data regarding OS during pregnancy. This interpretation did not take metabolic demands, prooxidant levels, the status of other antioxidants, or existing damage due to ongoing OS into consideration. It is important to note that measurement of OS in biological fluids may not be a true reflection of cellular level OS as it is difficult to measure. Redox balance is a coordinated function of multitudes of molecules, and examining antioxidants alone may not be sufficient to gage the intensity of OS. However, examining the OS-induced damage can generate better conclusions regarding OS than examining antioxidant levels alone. Figures 3C,D demonstrates the evidence for OS-induced generation of DNA and lipid peroxidation products (8-OxodG and F2-Isoprostanes, respectively). Our analysis showed that maternal plasma OS damage is higher in pPROMs than in both normal term births and PTBs. To note, the damages are minimal at term birth although labor and delivery are documented to have higher OS. It is expected that at term labor and delivery the metabolic demands are very high, and mature fetuses and fetal tissues of the intra uterine cavity generate OS and OS-induced signals from cellular damages. However, at term, a normal physiologic redox balance is fully functional and thus minimizes the OS-induced damage to cellular elements. Risk-induced OS and overwhelming damage generated signals are unlikely to be controlled by cellular or organ-level antioxidant capacity, leading to premature activation of signals for PTB or pPROM.

## Can OS Damage Lead to Adverse Pregnancy Outcomes?

Oxidative stress and OS-induced damage can produce a spectrum of genetic, metabolic, and cellular responses, and prooxidants exert their effects on cellular elements – namely lipids, proteins, and nucleic acids – disrupting their expression, structure, and function (46, 54, 62). Peroxidation of proteins leads to the loss of the sulfhydryl groups and linking of carbonyl groups with the side chains of other amino acids (63, 64). Although these proteins are normally proteolytically cleared from the system, under heightened ROS, oxidized proteins do not undergo proteolysis but rather accumulate as long hydrophobic bonds that affect cell function (63).

Cell membrane phospholipids are always a target of ROS activity (48). The peroxidized cell membrane stiffens, loses its selective permeability, and loses its integrity. Oxidized cell membranes are also susceptible to action by phospholipase enzymes that can activate a series of enzymatic and non-enzymatic breakdowns of oxidized phospholipids. One of the non-enzymatic by-products of this lipid peroxidation is F2-Isoprostane, which is considered the biomarker of ROS (65).

Reactive oxygen species causes the most lethal damage on DNA. This damage can result in single- or double-strand breaks, interchanging of sister chromatids, crosslinking of DNA to DNA or protein, or base modifications (66–68). Although all four bases of DNA are susceptible to ROS-mediated alterations, hydroxylated nucleotide 8-hydroxy-deoxyguanine (8-OHdG) was for the first time referred to as a major product of oxidative DNA damage (see Glossary), and high concentrations of 8-OHdG in biological fluids are considered a biomarker of ROS (69–80). Damages in DNA are constantly repaired, and simply measuring 8-OHdG levels is not sufficient to measure the extent of ROS; however, higher concentrations of 8-OHdG clearly indicate an underlying pathology that cannot be ignored.

## Documentation of OS Damage in PTB, pPROM, and Normal Term Birth

This section of the review explains some of the ongoing basic research studies designed to address OS-induced damages and how they differentially associate with PTB, pPROM, and normal term birth (81, 82). Briefly, we examined amniotic fluid samples for OS markers (lipid peroxidation – F2-Isoprostanes) and OS-induced 3-nitrotyrosine modified proteins (3-NT) in fetal membranes from PTB, pPROM, and term birth. Both PTB and pPROM had higher amniotic fluid F2-Isoprostanes than term birth. Cigarette smoking during pregnancy was associated with higher F2-Isoprostanes, regardless of pregnancy outcomes, and infection (in PTB). It was also associated with higher F2-Isoprostanes, compared to PTB with no infection. 3-NT staining, a marker of protein modification by OS, was more similar between term birth and pPROM than PTB.

In an earlier study, we reported changes associated with telomere length, a marker of aging, and OS. Telomeres are DNA–protein complexes, consisting of 5–15 kbp of repetitive DNA sequences, located at the ends of the chromosomes. They are essential for chromosome stability and cell survival, protecting them from end-to-end fusion and degradation. With each cell division, telomeres are shortened, and once a critical shortening is attained, division cycles halt and cell senescence (see Glossary) is triggered. Thus, telomere lengths serve as a valid marker of a cell’s biologic age. OS can induce single and double DNA strand breaks, which are detrimental to telomere shortening. Therefore, telomere shortening can be caused by natural aging or pathologically induced premature aging due to OS.

Interestingly, telomeres are shorter in early pPROM (<34 weeks) than in gestational age-matched PTB with intact membranes. However, telomere lengths are similar between early pPROMs and term births (>40 weeks), and it is suggestive of a similarity between the two phenotypes of pregnancy (83). Several such similarities in molecular and histologic markers exist between pPROM and normal term birth that lead us to hypothesize that pPROM may have early aging fetal tissues that may be prompting rupture and delivery.

We have examined likely causes and mechanisms of telomere shortening in fetal tissues. The next sections of this review will describe how OS-induced damages and tissue level changes may generate signals of parturition during normal pregnancies and how they may be pathologic in pPROM and a subset of PTB.

## OS-Induced DNA Damage and Its Consequences

**Figure 4:**
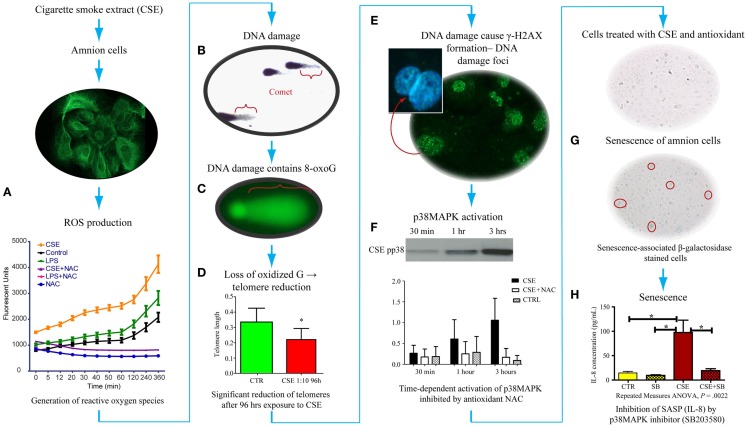
**Proposed pathways of senescence activation in human amnion primary epithelial cell cultures**. **(A)** Amnion cell cultures established from normal term Cesarean section placental membranes (cytokeratin stained) were exposed to cigarette smoke extract (CSE), and reactive oxygen species (ROS) generation was measured. ROS kinetics were monitored using 2′-, 7′-dichlorodihydrofluorescein diacetate (H2DCFDA) as an indicator for ROS in cells. Changes in DCF fluorescence were expressed as arbitrary fluorescence units. CSE-induced ROS generation was inhibited by N-acetyl cysteine. LPS also induced ROS but at a much lower level than CSE. **(B)** We performed two separate experiments to document: (1) DNA damage (Comet assay-silver staining) and (2) 8-OxoG production (FLARE) in amnion cells for Comet and FLARE. Amnion cells were harvested after treatment with CSE and immobilized in a layer of low melting point agarose on the FLARE slide. We demonstrated Comet formation after CSE treatment of amnion cells, confirming DNA damage with CSE. **(C)** Fragment length analysis using repair enzyme (FLARE) assays showed a similar Comet formation that confirmed 8-oxoG production, one of the most lethal biomarkers, in response to CSE, indicating 8-oxoG:OGG1 complex activity. **(D)** Exposure of these cells to ROS (by CSE) led to reduction of telomere length (*P* < 0.05), confirming a similar observation reported in fetal membranes from pPROM. **(E)** Telomere reduction by ROS can cause DNA damage foci (DDF) formation, indicating chronic OS damage. Amnion cells showed phosphorylated histone B protein (g-H2AX). **(F)** These amnion cells exposed to CSE also demonstrated a time-dependent activation of p38MAPK that persisted for 96 h. This activation was inhibited by NAC, confirming ROS-mediated effect. **(G)** p38MAPK (a pro-senescence protein) caused development of senescence-specific b-galactosidase staining in cells, confirming senescence of amnion epithelial cells after CSE exposure. Senescence development led to the production of one of the cytokines associated with senescence-associated secretory phenotype (SASP). **(H)** We confirmed that this is a SASP or p38-mediated effect when IL-8 was down-regulated after treating amnion cells with CSE and p38MAPK inhibitor (SB203580).

As detailed in previous sections, OS causes changes in DNA bases, especially Guanine. We have tested the generation of 8-OxoG formation in fetal amnion cells in response to OS. Figure 4 provides experimental evidence for DNA damage and its likely consequences. The OS response was induced in laboratory conditions using water soluble cigarette smoke extract (CSE) that is a known OS producer and a well-known risk factor of adverse pregnancy outcomes. Primary amnion cells obtained from placentas from normal term births, not Cesarean births, were used for this study (84). When exposed to CSE, amnion cells produced ROS (Figure 4A). This ROS production was down-regulated by antioxidant N-acetyl cysteine treatment, confirming OS production by amniocytes. These amnion cells also demonstrated significant DNA damage (Figure 4B), and the DNA lesions contained 8-OxoG (Figure 4C). High 8-OxoG levels may explain the shortened telomere length observed in a prior report, as these repetitive sequences are guanine rich and susceptible to ROS (83). To verify the shortening of telomeres, we treated the cells with CSE. CSE-induced telomere reduction was visible after 96 h compared to untreated controls, confirming that OS-inducing risk factors may cause oxidation of DNA that can lead to telomere attrition (Figure 4D). Telomere uncapping occurs when cells have critically shortened telomeres or when telomere-protective factors are impaired (85). Loss of telomere function can induce cell cycle arrest and senescence. One of the consequences of DNA damage and telomere attrition is also formation of DNA damage foci (DDF) in cells.

We have also analyzed the consequences of DNA damage and telomere attrition in amnion cells. DNA damage is a regular event during growth, and it is precisely fixed or repaired by multitudes of DNA repair mechanisms (86–92). However, persistent DNA damage in response to overwhelming OS or in response to OS-inducing risk factors, as often seen during adverse pregnancies, causes DNA breaks followed by the phosphorylation of the histone H2AX, which is a variant of the H2A protein family known as γ-H2AX. Analysis of amnion cells that lost telomere fragments after exposure to CSE revealed generation of DDF (Figure 4E). Signals arising from cells with DDF will eventually determine the fate of the affected cell through multitudes of pathways activating several cell cycle regulatory molecules (93–97). Analysis of multiple cell cycle regulatory factors revealed activation of p38 mitogen activated protein kinase pathway (p38MAPK) in amnion cells in response to CSE exposure (Figure 4F). p38MAPK activation by phosphorylation of its catalytic site residues, threonine-180 and tyrosine-182, promotes cell cycle arrest and cellular senescence by targeting the expression of proteins of SP (Figure 4G) (98, 99). Activation of p38MAPK to phosphorylated p38MAPK (pp38MAPK) by SP was prevented by antioxidant NAC confirming the influence of OS in p38MAPK activation. It is interesting to note that we did not see activation of another pro-senescence protein p53 in amnion cells treated with CSE although this has been reported in maternal decidual cells in animal models of pregnancy by SK Dey’s group.

## Activation of Senescence in Fetal Cells by p38MAPK

p38 Mitogen activated kinase is a pluripotent molecule and one of its functions is to activate senescence (aging) of the cell. Senescence is characterized by irreversible arrest of cell growth (99–103). Irreversible growth arrest at the G1 phase of the cell cycle is a major characteristic of senescence. Unlike apoptosis, these cells persist, alter their function, and change the tissue environment, inducing a unique signature of inflammatory markers similar to those seen in PTB and pPROM (104, 105). The amniochorionic cells divide at a rapid rate throughout gestation to accommodate the increasing demands of the intrauterine contents. This proliferative activity is a normal process that is expected to continue even at term (106). Overwhelming OS forces cells to undergo transformation either as malignant or senescence (107). Hostile pregnancy environments promote senescence of membrane cells and activation of an inflammatory condition that we propose causes adverse pregnancy outcomes as seen predominantly in early pPROM and a subset of PTBs complicated by chronic OS. We were able to document senescence of amniotic cells exposed to CSE using senescence-associated β-Galactosidase (SA-β-Gal) staining (Figure 4G). The number of SA-β-Gal positive cells was significantly higher after CSE treatment than untreated control amnion cells, confirming the development of senescence phenotype. Morphologic examination using transmission electron microscopy revealed features of senescence in fetal membrane cells, characterized by enlarged cells with senescence-associated heterochromatic foci, enlargement of organelles, and particularly endoplasmic reticulum and mitochondria. Senescing cells are also reported to produce a unique inflammatory signature known as senescence-associated secretory phenotype (SASP) (see Glossary) (104, 108, 109). SASP markers include cytokines, chemokines, growth factors, angiogenic factors, MMPs, and tissue inhibitors of matrix degrading enzymes, adhesion molecules, receptors, and receptor antagonists. Many SASP factors are reported as inflammatory mediators of pregnancies, and therefore it is not surprising to see more of them during preterm and term labors. Treatment of amnion cells with CSE and p38MAPK inhibitors (SB203580) substantially reduced IL-8 to the level of controls (Figure 4H). IL-8 generation as a part of CSE treatment and controlling its production through p38MAPK pathways suggests that the inflammatory process associated with term labors, preterm labors, and pPROMs can also arise in the absence of infections, and it is likely generated by senescing cells of the placenta and fetal membranes. Based on these findings and ongoing research in the laboratory, I propose that the source of inflammation is not just invading immunocytes, but it is likely that senescing fetal cells generate signals of parturition at term. In pPROM or PTB, a pathologic trigger resulting in OS can cause premature fetal cell senescence and SASP signaling onset of labor or cause rupture.

## Differences between PTB and pPROM; Can OS Determine Outcomes?

As described in previous sections, there are several similarities between early pROM and term birth. Similarly, several salient differences exist between PTB and pPROM although they have epidemiological and clinical similarities. Many of the similarities and differences are listed in Table 2. Inflammation is an underlying factor in PTB and pPROM; however, the extent of OS damage may be higher in pPROM, especially those that are early (<34 weeks of gestation). OS and inflammation are inseparable events, and biomarkers executing these responses are linked. It is also important to note that PTB is not devoid of any of the markers or indications listed in Table 2. In comparison with pPROM, the degree of OS and proteolysis seems to be more minimal in PTB than in pPROM. These findings are limited to early PTB and pPROM. Data are more inconclusive in cases between 34 and 37 weeks of gestation, late PTB, and pPROM. Based on the data used in *in vitro* models and *in situ* findings, pPROM seems to result from chronic OS whereas PTB results from acute OS. Chronic and overwhelming OS can cause telomere attrition, DDF formation, p38MAPK activation, fetal cell senescence, and SASP whereas acute OS may be sustainable by cells’ ability to resist OS-induced damages but still develop an inflammatory environment that is different than SASP. The former is expected to cause pPROM, and the latter may likely lead to PTB. In light of this evidence, it is also possible that infection is not the causal factor for a large subset of pPROMs and PTBs. Non-infectious cellular atrophies due to OS or OS-induced senescence cause sterile inflammation that can generate an immunocompromised intrauterine setting. A recent report by Romero et al. supports the concept of sterile intra-amniotic inflammation (110). When a sterile inflammatory milieu is created, it may provide an ideal environment for microbial invasion or activation of resident microbial flora in which case infection is likely secondary to an underlying pathology.

**Table 2:** **Markers studied in various maternal–fetal compartments and their similarities and differences between PTB with intact membranes and preterm premature rupture of the membranes (pPROM)**.

Biomarker	Sample	Indication	pPROM vs. PTB
F2-IsoP	Amniotic fluid	Oxidative stress	Higher in pPROM
3-NT staining	Fetal membranes	Oxidative stress	Higher in pPROM
Salivary proteases	Maternal saliva	Proteolysis	Higher in pPROM
MMPs/TIMPs	Amniotic fluid	Proteolysis	Higher in pPROM
Cytokines (IL-1, TNF, IL-6, IL-8)	Amniotic fluid	Inflammation	No difference
Telomere length	Fetal DNA and fetal membranes	Senescence	Higher in PTB
p38MAPK	Fetal membranes	Senescence	Higher in pPROM
p53	Fetal membranes	Senescence	Higher in pPROM
8-OxodG	Maternal plasma	DNA damage	Higher in pPROM
8-Oxoguanine glycosylase (OGG1)	Fetal membranes	DNA damage repair	Higher in PTB
Ras-GTPase	Fetal membranes	Intracellular signaling	Higher in PTB
Fas/Fas L	Fetal membranes	Apoptosis	Higher in pPROM

**Figure 5:**
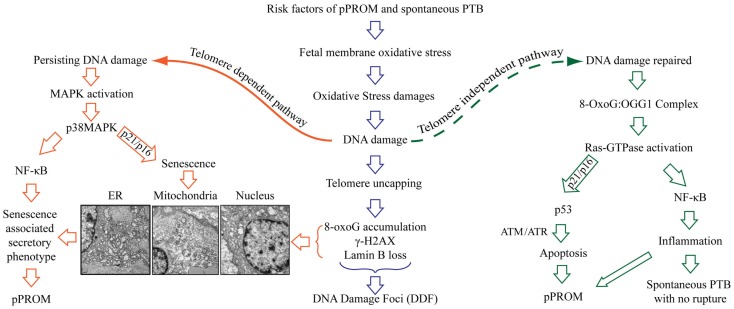
**Oxidative stress-induced pathways of pPROM and spontaneous PTB with intact membranes**. Risk factors of pPROM and PTB – like infection, obesity, poor nutrition, stress, behavioral risk factors (cigarette smoking, drinking, and drug abuse) – can cause oxidative stress (OS) in human fetal membranes that leads to OS-induced damages to all major cellular elements (lipids, proteins, and nucleic acid). Damages to DNA are especially lethal to fetal membranes and placental cells because they lead to oxidation of guanine base, resulting in mutagenic 8-OxoG formation. Telomeres are rich in G bases, and oxidation G bases lead to telomere attrition as a part of damage repair [base excision repair (BER)]. The pathway of pPROM and PTB may be delineated at this stage based on the type of risk and the type of OS response: overwhelming OS (chronic OS with smoking, alcohol use, obesity, poor nutrition, etc.) vs. infection/inflammation-related acute OS by fetal cells or immunocytes. Overwhelming OS can lead to persistent DNA damage and telomere reduction, 8-OxoG formation, resulting in DNA damage foci (DDF) formation. Telomere-dependent pathway – when OS is too high to be controlled by antioxidant mechanisms of the intrauterine tissues, especially fetal membranes, a telomere-dependent pathway arises where either OS itself or damaged DNA and other cellular elements can trigger p38MAPK activation either through NF-κB activation or direct cellular senescence. This is characterized by rounded, swollen organelles, and nuclear condensates. Additionally, senescing cells generate a unique set of biomarkers (senescence-associated secretory phenotype – SASP). SASP is characterized by cytokines, chemokines, matrix metalloproteinases, growth and angiogenic factors, and eicosanoids, all of which are involved in promoting labor. Telomere-independent pathway – in this pathway, oxidized Gs are excised from the damaged region by 8-oxoguanine glycosylase (OGG1) as a part of BER. 8-OxoG:OGG1 complex then activates Ras-GTPase and either promotes p53-mediated apoptosis or NF-κB-mediated inflammation. The exact mechanism of this switch is still unclear. Apoptosis is previously linked to pPROM. The latter pathway of NF-κB-mediated inflammation can be linked mostly to PTB with intact membranes.

The delineation of PTB and pPROM pathways depends on several factors and are not limited to the: (1) type and load of risk factors, (2) antioxidant status of subject, (3) gestational age, (4) overall immune status of the individual, or (5) genetic, epigenetic, and other socio-demographic factors. Based on the data described above, a new model of PTB and pPROM delineating pathways are described in Figure 5. These cases can be divided into subsets based on OS/inflammation and OS/inflammation-induced damages; therefore, overlap between subsets are expected and rupture or lack of rupture in PTB may depend on an individual’s profile as listed in the above paragraph. OS-induced DNA damage can cause telomere uncapping; however, depending on the strength of ROS and redox imbalance, pathways can arise in a telomere-dependent and independent way. Telomere-dependent pathways will result if base excision repair mechanisms fail to restore the damaged segments and the cells proceed to undergo cell cycle arrest and eventual cell death. Data from our laboratory demonstrate classic signs of telomere attrition and telomere damage-induced changes (8-oxoG accumulation, γ-H2AX, and nuclear membrane structure related Lamin B loss) in pPROM membranes or in amniotic fluid (data not shown). Many of these factors are either minimal or non-existent in a majority of PTB cases. Telomere-independent pathways can arise where DNA damage is minimal or where base excision repair mechanisms are functional and able to rebuild the damaged segments. During this process, 8-OxoG generated due to oxidative damage of telomere segments or G bases in other parts of DNA are repaired by a specific enzyme called 8-Oxoguanine glycosylase (OGG1) (88, 111–113). Recent findings suggest that 8-OxoG:OGG1 complex can cause Ras-GTPase activation, resulting in inflammation (114–116). Fetal membrane analysis of OGG1 mRNA expression also revealed more OGG1 in PTB than in pPROM, suggesting that DNA repair is more active in PTB than in pPROM due reduced availability of OGG1 (117). In unpublished findings, we also noticed higher Ras-GTPase activation in fetal membranes from PTB than pPROM. Although we have not confirmed this descriptive data through mechanistic studies, it is also possible that telomere-independent activation of DNA damage repair may cause two separate events as shown on the right side of Figure 5.

8-OxoG:OGG1 complex (DNA damage repair) can cause Ras-GTPase activation culminating in either antitumor p53 (proapoptotic) activation, resulting in pPROM, or culminating in master transcriptional factor NF-κB activation, resulting in inflammation without apoptosis. Earlier reports have shown evidence of apoptosis in fetal membranes from pPROMs through p53 pathways (118–123). Therefore, it is likely that both apoptosis and senescence may be seen in pPROM and will be a subset (based on exposure and host response). It is also important to note that we did not document active p53 in membranes from pPROM in our studies reported a decade ago. However, we did see effector caspase activation suggesting that apoptosis and senescence may both have independent roles in pPROM outcomes. NF-κB activation and its contribution are well reported by several investigators (124–130). NF-κB activation can occur by multitudes of risk factors’ specific signaling routes in PTB and pPROM or by maternal–fetal endocrine signals of initiation of parturition at term. DNA damage repair associated signals may also cause NF-κB activation resulting in PTB and pPROM, but this is not a well-studied mechanism in PTB and pPROM.

## Can Fetal Cell Senescence Trigger Term Birth?

Senescence of fetal membrane cells are very pronounced in term (>40 weeks) deliveries but not in term Cesarean sections when the patient is not in labor. Fetal membranes and placentas experience considerable OS at term prior to initiation of labor, which can lead to p38MAPK activation similar to that seen in pPROM membranes, causing senescence and further enhancement of SP and SASP. Fetally derived SASP factors are signals of maturation and can promote labor at term. The inflammatory milieu observed at term is of non-infectious origin, and senescence/SASP is likely one of the factors contributing to this phenomenon. Ongoing work in our research laboratories will further elucidate this mechanism, and understanding this phenomenon will improve our knowledge of the labor process and likely allow us to develop screening and diagnostic markers and identify intervention targets based on OS leading to p38MAPK and senescence.

## Summary

Oxidative stress is an inevitable component of pregnancy, and it is tightly regulated until labor and delivery. OS build-up at term due to an increasing demand from the fetus and heightened physiologic stress promotes senescence. Fetal tissues, placentas, and maturing fetuses generate signals of aging prompting labor and delivery. These signals we report here as SASP are mediators of uterotonic activities. These SASP signals may very well be regulated or influenced or in tandem with already reported biologic, endocrine regulated mechanisms of labor triggers (131–135). Risk factors of adverse pregnancies may cause premature aging of fetal tissues, triggering pathological mechanisms (specifically pPROM and in a subset of PTB with high OS damage as demonstrated by our data) that may result in premature activation of labor and/or rupture of the membranes. Reduction of adverse outcomes requires better characterization of biomolecules and pathways to understand their precise roles in triggering premature aging.

## Conflict of Interest Statement

The author declares that the research was conducted in the absence of any commercial or financial relationships that could be construed as a potential conflict of interest.

## Glossary

### Oxidative damage

Reactive oxygen species generated due to various physiologic and pathologic processes can lead to damage of major cellular elements, including DNA, RNA, proteins, and lipids, causing dysfunctional molecules that impact cellular integrity. Oxidative stress damage is associated with several disease processes and is also seen in the natural aging process.

### Oxidative DNA damage

Reactive oxygen species often target the DNA and cause oxidatively damaged DNA base lesions. Guanine is most prone to have oxidative damages, primarily 8-oxo-7, 8-dihydroguanine (8-oxoG). Telomeres, the chromosomal protective caps, are rich in Guanine bases, and they succumb to oxidative damage. Telomere attrition, seen in certain pregnancy complications, denotes oxidative stress during pregnancy complications.

### Senescence

Senescence or biologic aging involves the phenomenon of irreversible arrest of cell growth. Unlike apoptosis, these cells persist, alter their function, and change the tissue environment, inducing a unique signature of inflammatory markers. Placental and fetal tissues at term experience intensified oxidative stress and cellular damage, resulting in senescence. Senescent fetal cells can be considered as fetal signals of maturation to initiate labor.

### Senescence-associated secretory phenotype

Cells undergoing senescence produce a distinctive tissue microenvironment that transforms these cells into inflammatory cells characterized unique biochemical markers. These markers include cytokines, chemokines, growth and angiogenic factors, matrix degrading enzymes, and inhibitors among other classes of proteins. Fetal cell senescence can cause senescence-associated secretory phenotype (SASP) production with uterotonic properties and acts as signals of labor initiation.
